# A New Species of *Mimosa* L. ser. *Bipinnatae* DC. (Leguminosae) from the Cerrado: Taxonomic and Phylogenetic Insights

**DOI:** 10.3390/plants9080934

**Published:** 2020-07-24

**Authors:** Matías Morales, Renée H. Fortunato, Marcelo Fragomeni Simon

**Affiliations:** 1 Instituto de Recursos Biológicos, CIRN–CNIA, Instituto Nacional de Tecnología Agropecuaria (INTA), Hurlingham 1686, Buenos Aires, Argentina; fortunato.renee@inta.gob.ar; 2 Consejo Nacional de Investigaciones Científicas y Técnicas (CONICET), Ciudad Autónoma de Buenos Aires C1425FQB, Argentina; 3 Facultad de Agronomía y Ciencias Agroalimentarias, Universidad de Morón, Morón B1708JPD, Buenos Aires, Argentina; 4 Embrapa Recursos Genéticos e Biotecnologia. Parque Estação Biológica, Brasília, DF 70770-917, Brazil; marcelo.simon@embrapa.br

**Keywords:** Brazil, Cerrado, Fabaceae, Maranhão, morphology, taxonomy, *trnD-trnT*

## Abstract

*Mimosa carolina* (Leguminosae), a new species from the Parque Nacional Chapada das Mesas, located in the northern limit of the Brazilian Cerrado, is described and illustrated. In addition, a phylogenetic reconstruction was performed to recover the position of this species in relation to the main clades of the genus *Mimosa.* This new species is assigned to sect. *Habbasia* ser. *Bipinnatae* and exhibits relevant morphological differences with all described species of this series, most notably the prostate habit, glabrous stems, and absence of internal spicules. Our results indicate that this new entity is clearly nested in a strongly supported clade with other striated-corolla species of ser. *Bipinnatae.* Therefore, ser. *Bipinnatae* appears to be monophyletic, and a morphologically and ecologically cohesive group within *Mimosa.* An updated identification key for this series is provided.

## 1. Introduction

The large genus *Mimosa* (Leguminosae) is particularly diverse in the Cerrado, a savanna-dominated ecoregion covering 2 million square kilometers in central Brazil and eastern parts of Bolivia and Paraguay [1,2]. Vegetation in the Cerrado varies from tall savanna woodlands to open grasslands, with gallery forests or seasonally waterlogged marshes and fields occurring along watercourses. Soils are generally nutrient-poor, highly acidic and aluminum-rich, and precipitation is highly seasonal with a pronounced winter dry season from May to October [2]. High seasonality and the accumulation of dry biomass makes Cerrado savannas and grasslands fire-prone, and species from many plant lineages show adaptations to cope with fire regimes [3].

The detailed monograph of Barneby [4] for the Neotropics and latter studies [5,6,7,8,9,10,11,12] allowed the recognition of ca. 200 species of *Mimosa* in the Cerrado. Most of these species exhibit a restricted distribution; consequently, this ecoregion exhibits high levels of endemism for the genus, particularly in the highlands [13].

This genus has been divided into five sections, largely based on floral and trichome characters [4]: sect. *Mimadenia* Barneby, sect. *Batocaulon* DC., sect. *Habbasia* DC., sect. *Calothamnos* Barneby, and sect. *Mimosa.* However, new phylogenetic insights are challenging this proposal; in these works, based on different chloroplast markers [14,15], only sect. *Mimadenia* was recovered as monophyletic. Members of sections *Batocaulon* and *Habbasia* formed a paraphyletic clade, whereas sect. *Calothamnos* was nested within sect. *Mimosa*.

In spite of the discussion about the sections proposed by Barneby [4] several infrageneric groups of the genus at the level of series or subseries were recovered as monophyletic, such as sect. *Batocaulon* ser. *Stipellares* Benth., sect. *Mimosa* subser. *Obstrigosae* (Benth.) Barneby, and sect. *Habbasia* ser. *Bipinnatae* [14,15]. However, for many infrageneric groups, taxonomic sampling needs to be increased to allow for more robust conclusions.

Detailed studies to infer diagnostic characters for well-supported groups in *Mimosa* are still lacking. A preliminary assessment of character evolution in *Mimosa* has shown that, except for extrafloral nectaries and pollen grain morphology, the main characters used to define infrageneric groups are homoplasic [15]. The fact that many morphological characters evolved independently in different clades makes the search for diagnostic characters a challenging task in *Mimosa*.

The aim of this work was to evaluate a possible new *Mimosa* species from the Cerrado, as well as investigate its phylogenetic position in the genus [15]. In addition, we re-evaluated the monophyly of ser. *Bipinnatae* with the inclusion of newly generated sequences in the phylogeny.

## 2. Results

### 2.1. Taxonomic Treatment

**Figure 1:**
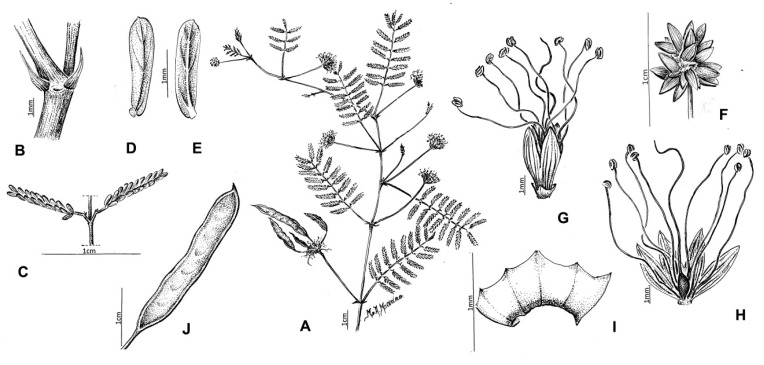
*Mimosa carolina.* (**A**) Flowering and fruiting branch; (**B**). Stipules. (**C**). Section of the primary rachis with one pair of pinnae. (**D**). Leaflet, adaxial face. (**E**). Leaflet, abaxial face. (**F**). Inflorescence. (**G**). Closed flower. (**H**). Opened flower. (**I**). Opened calyx. (**J**). Fruit. Drawn from the isotype (BAB) by Angélica Marino.

**Figure 2:**
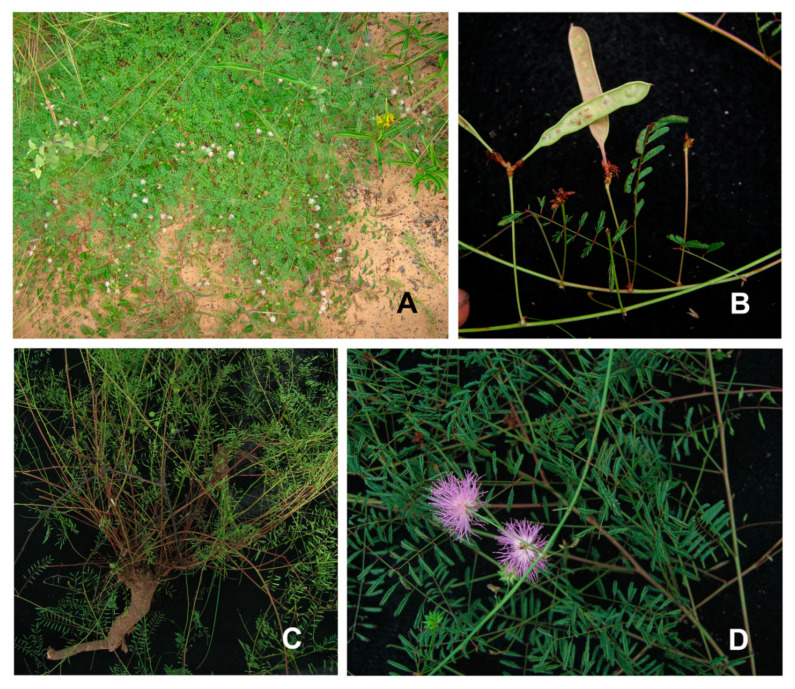
*Mimosa carolina.* (**A**). Prostate habit. (**B**). Immature fruits. (**C**). Base of plant with several slender branches arising from a wood xylopodium. (**D**). Inflorescence. Photos by Marcelo Simon.

*Mimosa carolina* M.Morales & Marc.F.Simon, *sp. nov*. Figure 1 and Figure 2.

#### 2.1.1. Type and Diagnosis

Brazil. Maranhão: Carolina, Parque Nacional da Chapada das Mesas, acesso N no km 519 da BR-230, 10 km N, Gleba 2, 09 April 2016, *M. F. Simon* et al. *2828* (holotype: CEN!; isotypes: BAB!; HUEFS!; IAN!; RB!).

*Mimosa carolina* is similar to the widespread *M. somnians* Humb. & Bompl. ex Willd, from which it differs by a prostrate habit with slender stems (vs. erect subshrub with robust stems), that are glabrous (vs. pubescent to hispid, rarely glabrous), and unarmed (vs. sometimes armed), shorter pinnae (4–8 mm long) with 6–10 pairs of leaflets (vs. pinnae 10–55 mm long with 13–50 pairs of leaflets), the insertion of pinnae on leaf rachis v-shaped (vs. insertion of pinnae straight on the leaf rachis), and the absence of interpinnal spicules (vs. interpinnal spicules present); it differs from the poorly known *M. leptorhachis* Benth. by the prostrate habit (vs. erect), glabrous stems (vs. hispidous), insertion of pinnae on leaf rachis v-shaped (vs. insertion of pinnae straight on the leaf rachis) and absence of interpinnal spicules (interpinnal spicules present). In addition, it differs from *M. brachycarpa* Benth. by its prostrate habit (vs. erect 1–2 m tall), petioles which are 25–45 mm long (vs. 1–10 mm long) and larger (36–40 mm long), and glabrous fruits (vs. fruits 12–18 mm long, densely setose).

#### 2.1.2. Description

Procumbent subshrubs, with woody, napiform xylopodium. Stems slender, creeping, unarmed, glabrous. Stipules 1.5–2.8 × 0.1–0.2 mm, ovate and mucronate or aristate, glabrous, one-nerved (sometimes three but tenuous), persistent; petioles 6–21 mm long, terete or subterete; leaf rachis 25–45 mm long, including petiole 8–16 mm long; pinnae (3) 4–8–pairs, rachis 4–8 mm long; leaflets 6–10–pairs, 1.5–3 × 0.5 mm, mainly oblong, glabrous, the midrib branched beyond the middle, tenuously 1–3 nerved or nerveless. Inflorescences axillar, with peduncles 20–27 mm long; capitula 3–6 mm in diameter, moriform or stelliform; floral bracts 0.5–1 × 0.1 mm, lanceolate, glabrous, 1–nerved, homomorphic, not overpassing the flowers before the anthesis, hardly persistent at anthesis. Flowers tetramerous, diplostemonous; calyx 0.4–0.6 mm long, campanulate, denticulate, glabrous; corolla 2.5–2.75 mm long, lobes striately 7–10-nerved, glabrous overall, pale pink; filaments 6–7 mm long, free to minimally fused less than 0.5 mm long around the ovary, pink, anthers 0.3–0.4 mm long; ovary glabrous, style 6–7 mm long, glabrous, stigma poriform. Pods 36–40 × 3–4 mm, oblong to slightly oblanceolate, glabrous, 3–6-seeded, stipe up to 7 mm long, replum 0.3 mm wide, straight. Seeds not seen.

#### 2.1.3. Distribution and Ecology

**Figure 3:**
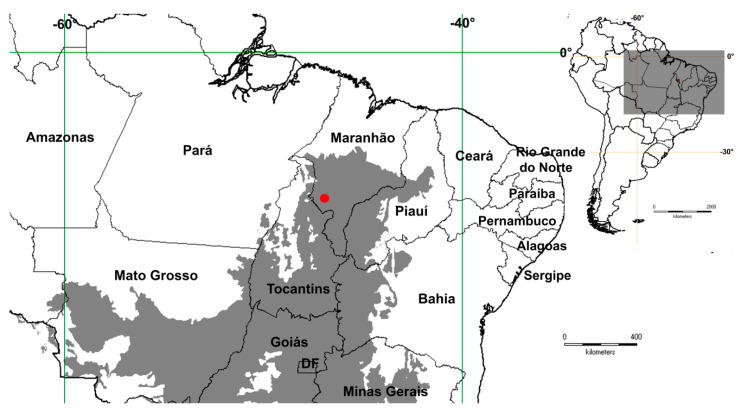
Geographic distribution of *Mimosa carolina* in northeastern Brazil. The dark gray area in the large map represents the Cerrado ecoregion.

At present, this species is only known from the Parque Nacional Chapada das Mesas, in Maranhão (Brazil), in the northern extreme of the Cerrado (Figure 3). This region has not been adequately prospected by botanists [16]; it explains why this entity has remained unknown. It was found growing on deep, sandy soils in sparse savanna vegetation at 280 m elevation, about sea level.

#### 2.1.4. Etymology

The epithet refers to Carolina, the municipality of Maranhão State in Brazil where the collection site is located.

#### 2.1.5. Conservation notes

*Mimosa carolina* is only known from the type locality at the Parque Nacional Chapada das Mesas (160,000 hectares). It was recorded as locally abundant in a collecting site in the southern portion of the national park (M. Simon pers. observation). According to the revision of specimens in herbaria (Supplementary material) in recent field trips, this species was not found in other localities. Considering that the region is poorly collected, we prefer to classify *M. carolina* as Data-Deficient (DD) according to the IUCN criteria [17].

### 2.2. Morphological and Phylogenetic Analyses

The taxonomic identification and morphological characterization of a set of more than 100 specimens from ser. *Bipinnatae* (Supplementary material) allowed the recognition of a new entity among recent collections from Maranhão State in Brazil. This new entity, *M. carolina*, was assigned to ser. *Bipinnatae* based on the presence of diplostemonous, tetramerous flowers with a plurinerved and striate corolla. This new species exhibits differences in several characters compared with other members of the ser. *Bipinnatae* (Table 1). It can be recognized by a combination of characters, some of them shared with other species from ser. *Bipinnatae*: prostrate habit with slender stems growing from a woody xylopodium; stems glabrous, unarmed; pinnae rachis 4–8 mm long bearing 6–10 pairs of leaflets; insertion of pinnae on leaf rachis v-shaped; and absence of interpinnal spicules (Figure 1 and Figure 2).

Other differences with species of *Bipinnatae* are: (1) from *M. brachycarpa,* by the lax foliage and leaves with longer petioles (vs. crowded foliage along stems and mainly subsessile leaves) and a higher number of ovules; (2) from *M. monacensis* Barneby and *M. poculata* Barneby by its homomorphic floral bracts (vs. heteromophic bracts and a collar of wider bracts at the basis of the capitulum); (3) from *M. surumüensis* Harms and *M. calliandroides* Hoehne, by the glabrous stems (vs. arborescent plumose–scabrous trichomes or scalelike trichomes); (4) from *M. microcephala* Humb. & Bonpl. ex Willd., *M. brachycarpoides* Barneby and *M. scaberrima* Hoehne by its plurinerved, striate corolla lobes (vs. 1–3-nerved) (Figure 1). We performed a key for identification of all species of the series *Bipinnatae* (Table 1).

**Figure 4:**
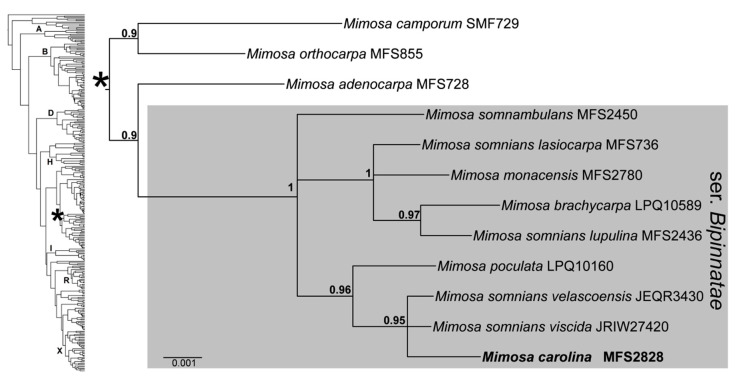
Phylogeny of members of ser. *Bipinnatae* including the new species *Mimosa carolina* based on a Bayesian analysis of the plastid *trnD-trnT* locus. Numbers above nodes of the majority rule consensus tree are posterior probability values (PP). On the left, a scheme of the phylogeny of the genus *Mimosa* from Simon et al. (2011) showing the main clades (letters) and approximate position (asterisk) of the clade shown in the right panel.

The present phylogenetic analysis, based on the plastid *trnD-trnT* region, included nine species, two subspecies and four varieties of ser. *Bipinnatae*, as well as three species from other series of sect. *Habbasia* as outgroups. The analysis recovered ser. *Bipinnatae* as a strongly supported clade (PP = 1), with *M. carolina* deeply nested within it (Figure 4). The sequence of *M. carolina* diverged from its closest relatives *M. somnians* var. *velascoënsis* (Harms) Barneby and *M. somnians* var. *viscida* (Willd.) Barneby by two nucleotide substitutions plus a six-nucleotide insertion. Sequence variation between the four taxa of *M. somnians* sampled ranged from a single insertion between vars. *velascoënsis* and *viscida*, to two insertions and five substitutions between vars. *lupulina* and *velascoënsis*.

## 3. Discussion

Based on morphological evidence, *Mimosa carolina* could be readily assigned to ser. *Bipinnatae* because of the presence of a striate corolla, a distinctive characteristic that defines this group [4], with few exceptions. *M. carolina* can be recognized by a combination of characters, some of them shared with other species from this series: a prostrate habit with slender stems radiating from a xylopodium; stems glabrous, unarmed; pinnae 4–8 mm long with 6–10 pairs of leaflets, insertion of pinnae on leaf rachis articulated forming a v-shape, and the absence of interpinnal spicules. The habit and absence of interpinnal spicules are two character states that seem to be exclusive to *M. carolina*, since most species of ser. *Bipinnatae* are erect subshrubs without prostrate stems, and interpinnal spicules are present in all species of the series [4]. The singular shape of the pinnae rachis of *M. carolina*, forming a V-shape after the pulvinule, is not found in the majority of taxa of ser. *Bipinnatae,* excepting *M. somnians* var. *leptocaulis* (Benth.) Barneby; the latter has a pinna rachis that forms a little angle after its insertion [4].

Although *M. leptorhachis* (erroneously annotated as “*Mimosa leptorachis*” by Barneby [4] (p. 463)) resembles *M. carolina,* the observation of the type of the former allowed us to consider them different species, due to the erect habit with robust stems and presence of interpinnal spicules in *M. leptorhachis.* In fact, the identity of *M. leptorhachis* is doubtful since it is only known from the type collection, which lacks carpological information [4,18], and no more specimens appear to be available in herbaria to allow a proper assessment of its rank and status.

Comparisons of sequence variation based on the *trnD-trnT* locus shows that *M. carolina* is genetically distinct from closely related taxa, reinforcing its classification as a separate species. The results of our phylogenetic reconstruction show that the new species is nested with all other representatives of ser. *Bipinnatae*, which formed a highly supported clade (PP = 1), in line with the morphological classification of Barneby [4]. Our increased sampling within this group (nine additional taxa) confirms previous results based on a smaller sampling (three species; [15]) and reinforces ser. *Bipinnatae* as a phylogenetically and morphologically cohesive group. This consistent infrageneric group now contains 13 species and 15 varieties, including taxa listed by Barneby [4], as well as *M. carolina*, described here. This series is distributed mainly in the Brazilian Planaltine and Guayana Highlands; only *M. somnians* has a wider distribution area, ranging from southern Mexico to northeastern Argentina [4]. Here, we did not include *M. trinerva* V.F.Dutra and F.C.P.Garcia, which was provisionally assigned to ser. *Bipinnatae* [6], since analysis of herbarium specimens indicates that it would be more properly placed in ser. *Pachycarpae* Barneby.

Our new phylogeny also included samples of four infraspecific taxa of *M. somnians*, which did not form a monophyletic clade. The divergence between infraspecific taxa of *M. somnians*, which appeared in different, well-supported clades, suggests that they might be better interpreted as distinct species. Indeed, this species is morphologically highly diverse and configures a taxonomic complex with four subspecies and ten varieties [4]; some of them were originally described as species [17] but later treated as varieties by Barneby [4].

The differentiation between infraspecific taxa of *M. somnians* in the literature has been based, in some cases, on quantitative traits with substantial overlap, making it difficult to separate them (e.g., *M. somnians* subsp. *longipes* (Barneby) Barneby; [4]). In other cases, diagnostic characters are discrete and allow a sharp differentiation. This is the case of *M. somnians* var. *lupulina* (Benth.) Barneby, which is easily separated from other varieties of *M. somnians* by its dilated bracts [4]. Incidentally, the close relationship between *M. somnians* var. *lupulina* and *M. brachycarpa*, which were recovered in our phylogeny as sister species, is supported by the presence of a set of wide external floral bracts in the inflorescence, which is shared by both taxa.

Overall, our phylogenetic analysis contributed to the understanding of the relationships between ser. *Bipinnatae* taxa, showing that *Mimosa* species with striate, plurinerved corollas comprise a genetically cohesive group, and infraspecific taxa under *M. somnians* do not form a monophyletic clade and their taxonomic status deserves future investigation. However, a more representative taxon sampling will be needed to better access relationships within ser. *Bipinnatae*, since only nine out of the 27 currently recognized taxa [4,7] have been sampled to date. Likewise, investigating species boundaries in more detail would require multiple accessions for each species and sequencing of highly variable loci that allow discrimination between species.

It is interesting to point out that the Chapada das Mesas National Park is one of the largest units of conservation in the Cerrado. Recent expeditions and taxonomic work resulted in the description of other endemic species from this location (e.g., *Philcoxia maranhensis* Scatigna, [19]; *Dyckia maranhensis* Guarçoni & Saraiva, [20]). Therefore, discovering new endemic species here reinforces the conservation value of this unit in the Biodiversity Hotspot of the Cerrado.

## 4. Materials and Methods

More than 100 specimens of ser. *Bipinnatae* from the following herbaria: BAB, CEN, CTES, LIL, MBM, MO, NY, RB, SI, SP, UB and UFG, were revised. This set of specimens comprised all taxa of the series covering their complete area of distribution (Supplementary material). The taxonomic identification was checked according to Barneby [4] and their morphological characters were measured and/or registered. This revision included the nomenclatural types of taxa and synonyms, as well as the images available from different herbarium databases: JSTOR [21], Kew Botanic Gardens [22], The Barneby Legume Catalogue [23], TROPICOS [24], and SpeciesLink [25].

Several field trips across the Cerrado in the Brazilian states of Goiás, Maranhão and Tocantins were carried out between 2016 and 2019 to collect specimens of ser. *Bipinnatae*. Samples of leaves for DNA extraction were also collected in these expeditions, which were dried in silica gel and stored at −18 °C in the EMBRAPA laboratory in Brasilia.

Taxon sampling for phylogeny (Table 2) included nine members of ser. *Bipinnatae* (including five newly sequenced taxa) plus three species (*M. adenocarpa* Benth., *M. camporum* Benth., and *M. orthocarpa* Spruce ex Benth.) selected as outgroups based on Simon et al. [15]. Seven sequences from previous studies [10,15] were retrieved from GenBank.

DNA was extracted using the protocol of Inglis et al. [26], which includes a pre-wash treatment with sorbitol to remove interfering metabolites. DNA was quantified using Nanodrop Nuclei Acid Quantification^®^ (Thermo Fisher, Waltham, MA, USA) and agarose gel to check integrity and concentration.

We based our phylogenetic analysis on the plastid *trnD-trnT* region, which was previously used to infer the phylogeny of *Mimosa* [10,15]. Amplification of the *trnD-trnT* region followed the same PCR protocol and primers (*trnD2*, *trnE*, *trnT*, and *trnY*), as described in Simon et al. [14]. Sequencing reactions using successfully amplified products were performed with the four primers using the Big Dye Terminator kit ver. 3.1 (Applied Biosystems, Foster City, CA, USA).

Consensus sequences from the four sequence strands were assembled using Geneious (v. 6.0.6, Biomatters Ltd.). Sequences generated in this work and those obtained from GenBank were aligned using Clustal W v. 2.1. [27] under default parameters. The aligned *trnD-trnT* dataset was composed of 12 terminals and 1495 bp. Bayesian analysis was carried out with MrBayes, version 3.2.2 [28], using the GTR + I + G nucleotide substitution model, which was the best model selected in jModelTest v. 2 [29].

We performed two runs in parallel of four Markov chain Monte Carlo for 10^6^ generations, with trees sampled every 5000 generations. Permutation of parameters was initiated with a random tree and four simultaneous chains set at default temperatures. Convergence of runs was assessed by inspecting whether the standard deviation of split frequencies of runs was less than 0.01, and the first 25% of the trees were discarded as burn-in. Trees sampled from post-burn-in were summarized into a 50% majority-rule consensus tree that included posterior probabilities (PP).

Voucher information, taxon authority, and GenBank accession numbers of newly generated sequences, as well as those of sequences published in other studies used in our analysis, are provided in Table 2. The aligned *trnD-trnT* dataset and trees generated in Bayesian analysis are available in the TreeBASE repository [30].

## Figures and Tables

**Table 1:** Key to Identify *Mimosa carolina* and Similar Species from Mimosa sect. Habbasia ser. Bipinnatae (Steps 2, 11 and 12 are adapted from Barneby [4]).

Dichotomy	Step/Species
1. Corolla 3–11–nerved	2
1′. Corolla 1–3–nerved	11
2. Setae of stems dilated and dorsiventrally compressed, scalelike	*M. calliandroides* Hoehne
2′. Setae of stems subterete, sometimes transversally dilated scarce overall the stems	3
3. Trichomes of indumentum branched, generally arborescent plumose–scabrous	*M. surumuënsis* Harms
3′. Trichomes unbranched	4
4. Leaflets up to 12–17 × 2–4.5 mm	*M. somnambulans* Barneby
4′. Leaflets up to 10 × 2 mm	5
5. Bracts of the lower flowers in each capitulum united into an involucre, generally heteromorphic	6
5′. Floral bracts homomorphic, not forming an involucre	7
6. Stems armed; pinnae constantly 2-pairs	*M. monacensis* Barneby
6. Stems unarmed; pinnae 2–4-pairs	*M. poculata* Barneby
7. Pinnae of leaves at mid-stem 2-pairs	*M. glaucula* Barneby
7′. Pinnae of leaves at mid-stem more than 2-pairs	*8*
8. Rachis of longer pinnae 4–8 mm long, and leaflets 6–10 pairs	*10*
9. Subshrubs humifuse, procumbent, with stems slender. Insertion of pinnae on leaf rachis notably articulated forming a v-shaped, interpinnal spicule absent	*M. carolina* M.Morales & Marc.F.Simon
9′. Subshrubs erect to procumbent with stems firm. Insertion of pinnae on leaf rachis notably not or barely articulated, interpinnal spicule 0.25–0.5 mm long	*M. leptorhachis* Benth.
10. Craspedia breaking only in valves, mostly 1–4-seeded. Ovules and seeds 1–4. Leaves subsessile with petiole 0.1–4 mm long	*M. brachycarpa* Benth
10′. Craspedia with typical dehiscence, breaking in articles. Ovules and seeds mainly more than 5. Leaves with petiole more than 5 mm long	*M. somnians* Humb. & Bonpl. ex Willd
11. Pinnae 7–30-pairs; cauline setae smooth and subterete, sometimes basally spurred	*M. microcephala* Humb. & Bonpl. ex Willd
11′. Pinnae 1–4-pairs; cauline setae scaberoulous	*12*
12. Cauline setae dilated, scalelike, lanceolate–triangular, basifixed; calyx ± one tenth as long as corolla	*M. scaberrima* Hoehne
12′. Cauline setae terete, spurred at base, thus laterally attached; calyx ± half as long as corolla	*M. brachycarpoides* Barneby

**Table 2:** Voucher information, locality, and GenBank accession numbers of taxa used in the phylogenetic analysis.

Taxon	Voucher (Herbarium)	Locality	GenBank Accession Number	Source
*Mimosa adenocarpa* Benth.	M.F. Simon 728 (CEN)	Brasília, Distrito Federal, Brazil	FJ981984	[15]
*Mimosa brachycarpa* Benth.	L.P. Queiroz 10589 (HUEFS)	Porto Estrela, Mato Grosso, Brazil	FJ982011	[15]
*Mimosa camporum* Benth.	S.M. Faria 729 (RB)	Oriximiná, Pará, Brazil	FJ982019	[15]
*Mimosa carolina* sp. nov.	M.F. Simon 2828 (CEN)	Carolina, Maranhão, Brazil	MT459463	This study
*Mimosa monacensis* Barneby	M.F. Simon 2780 (CEN)	Carolina, Maranhão, Brazil	MT459464	This study
*Mimosa orthocarpa* Spruce ex Benth.	M.F. Simon 855 (FHO)	Acayucan, Veracruz, Mexico	FJ982141	[15]
*Mimosa poculata* Barneby	L.P. Queiroz 10160 (HUEFS)	Oeiras, Piauí, Brazil	JF694269	[15]
*Mimosa somnambulans* Barneby	M.F. Simon 2450 (CEN)	Nova Roma, Goiás, Brazil	MT459460	This study
*Mimosa somnians* Humb. & Bonpl. ex Willd. var. *lasiocarpa* (Benth.) Barneby	M.F. Simon 736 (CEN)	São João D’aliança, Goiás, Brazil	FJ982194	[15]
*Mimosa somnians* Humb. & Bonpl. ex Willd. var. *lupulina* (Benth.) Barneby	M.F. Simon 2436 (CEN)	Niquelândia, Goiás, Brazil	MT459461	This study
*Mimosa somnians* Humb. & Bonpl. ex Willd. var. *velascoënsis* (Harms) Barneby	J.E.Q. Faria Jr. 3430 (CEN)	Cáceres, Mato Grosso, Brazil	MT459462	This study
*Mimosa somnians* Humb. & Bonpl. ex Willd. var. *viscida*	J.R.I. Wood 27420 (K)	Velasco, Sta. Cruz, Bolivia	KJ802912	[10]

## Supplementary Materials

The following are available online at https://www.mdpi.com/2223-7747/9/8/934/s1.
