# Supplementary material for: A New Species of Mimosa L. ser. Bipinnatae DC. (Leguminosae) from the Cerrado: Taxonomic and Phylogenetic Insights
Source: Plants (Basel). 2020 Jul 24;9(8):934. doi: 10.3390/plants9080934 (PMC7464646; doi:10.3390/plants9080934)
Supplement: Supplementary file 1 [file plants-09-00934-s001.pdf]

Supplementary Materials

5.1. Additional Selected Specimens Examined

5.1.1. *Mimosa brachycarpa Benth.*

BRAZIL. Goiás: Na Fazenda das Esmeraldas, perto Córrego Paciência e GO164, ca. 6 km NE of Goiás Velho, 12 Feb 1980, *J. H. Kirkbride* et al. *3418* (NY933075, UB). Minas Gerais: Montezuma, Sítio 13. Ponto 5, 18 Mar 2017, *A. C. Sevilha* et al. *6613* (CEN103595). Mato Grosso: Xavantina, 6 km E de Xavantina, 03 Aug 1967, *J. A. Ratter & J. Ramos 283* (UB); Xavantina, Km. 31 N of Xavantina on Cachimbo road, 01 Jan 1968, *D. Phylcox & A. Ferreira 3839* (UB); ca. 20 km S of Xavantina, 09 Jun 1966, *H. S. Irwin* et al. *16834* (UB); CA. 70 km S of Xavantina, 19 Jun 1966, *H. S. Irwin* et al. *17444* (UB); Xavantina–São Félix, 12 Oct 1968, *Sidney 1467 & E. Onishi 688* (UB). Piauí: Gilbués, 07 Jul 1995, *S. M. Rodrigues 531* (NY933095). Minas Gerais: Serra da Lapa, Nov 1824, *L. Riedel 17* (K, isotype). Tocantins: Filadelfia, 10 km W of Carolina, 07 Aug 1984, *G. T. Prance & N. T. Silva 58586* (NY933096).

5.1.2. *Mimosa brachycarpoides Barneby*

VENEZUELA. Bolívar: Ca. 30 km N of Puerto Ayacucho, 30 Jan 1975, *A. H. Gentry 14715* (NY, MO, isotypes); Carretera de Los Pijiguaos hacia Puerto Ayacucho, km 52, Serranía de Parguaza, al este de la carretera Bolívar, 21 Jan 1993, *A. Gröger 727* (N1345092).

5.1.3. *Mimosa calliandroides Hoehne*

BRAZIL. Amazonas: Humaitá, Estrada Humaitá–Jacarenanga, km 150, a 60 km ao Sul, 21 Jun 1982, *L. O. A. Texeira* et al. *1274* (NY01345156, MBM98047). Mato Grosso: Novo Aripuaná, Rod. do Estranho, km 120, entrada das minerações Igarapé Preto e São Francisco, 20 Apr 1985, *C. A. Cid Ferreira 5641* (MO2916514). Rondônia: Pouso [Primeiro] de Fevereiro, margens do Cantario, Feb 1919, *Kuhlmann 2025* (SP, holotype; RB, isotype).

5.1.4. *Mimosa glaucula Barneby*

BRAZIL. Bahia: Lagoa Itaparica, 10 km W of São Inácio – Xique – xique road at the turning 13, 1 km N of São Inácio, 26 Feb 1977, *R. M. Harley* et al. *19117* (CEPEC, holotype; NY, isotype). Tocantins: Goiatins, Estrada Graçolândia-Itacajá, margem esquerda do Ribeirão Cartucho, 04 May 2009, *G. Pereira-Silva* et al. *14395* (CEN99822).

5.1.5. *Mimosa leptorhachis Benth.*

Brazil. Minas Gerais: in deserto Frio versus flumen S. Francisco, no date, *Martius s.n.* (M, holotype seen in digital image).

5.1.6. *Mimosa microcephala Humb. & Bonpl. Ex Willd.*

5.1.6.1. *Mimosa microcephala* subsp. cataractae (Ducke) Barneby

5.1.6.1.1. *Mimosa microcephala* subsp. *cataractae* var. *cataractae*

VENEZUELA. Bolívar: Campamento El Salto, 22 Jun 1987, *E. Sanoja 1803* (NY1345908). FRANCE: French Guiana: Moint Saint-Marcel, 18 Jul 2002, *J. J. de Granville* et al. *15276* (NY1345911). BRAZIL.

Pará: In ripis fluminis Tapajoz prope cataractam Maranhão Grande, 25 Jun 1918, *A. Ducke 17070* (P, isotype, seen in digital image). Roraima: Rodovia BR-210, São Luis do Anauá, 26 Aug 1987, *C. A. Cid Ferreira* et al. *9231* (MBM233184).

5.1.6.1.1. *Mimosa microcephala* subsp. *cataractae* var. *lumaria* Barneby

GUYANA. Upper Takutú-Upper Esequibo, SE Kanuku, Mts. Makaparima, 4 km NNE of Crabwood Creek camp, 26 Oct 1991, *B. Hoffmann 410* (NY1345954). VENEZUELA. Amazonas: Vegetación a lo largo de Cañón Butrón, afluente del Caño Ucata, 21 Oct 1989, *G. A. Romero & E. Melgueiro 2149* (NY1345951). Bolívar: Hato La Vergareña, 3 km E of Cano Azul, 23 Oct 1954, *J. J. Wurdack & N. G. L. Guppy 178* (NY, holotype).

5.1.6.2. *Mimosa microcephala* subsp. *microcephala*

5.1.6.2.1. *Mimosa microcephala* subsp. *microcephala* var. *communis* Barneby

VENEZUELA. Bolívar: Alto Caroní, en quebradas de Río Ambetere, valle del Urimari, Jan 1949, *F. Cardona Puig 2587* (NY2911, holotype). COLOMBIA: Guainía: Immediately S of Casuarito, lajas along the Río Orinoco, 22 Jun 1984, *G. Davidse & J. S. Miller 26414* (NY1345947).

5.1.6.2.2. *Mimosa microcephala* subsp. *microcephala* var. *microcephala*

VENEZUELA. Amazonas. Atures, 8 km S of Puerto Ayacucho, Estación de Pisicultura de Puerto Ayacucho, 13-15 Apr 1978, *G. Davidse & O. Huber 14944* (NY1345966); Atures, alrededores de Puerto Ayacucho (±30 km al N, sabana de Rincones de Chacorro, 23 May 1979, *O. Huber 3788* (NY1345975).

5.1.6.2.3. *Mimosa microcephala* subsp. *microcephala* var. *mituënsis* Barneby

VENEZUELA. Amazonas: Ature, 22 km S of Puerto Ayacucho along road to Samariapo, near Garcitas, 16 Apr 1978, *G. Davidse & O. Huber 15142* (NY1345949). COLOMBIA. Vaupés: Cerro Mitú, 04 Sept 1959, *B. Maguire* et al. *44104* (NY, holotype).

5.1.7. *Mimosa monacensis Barneby*

BRAZIL. Goiás: Campos Belos, 2001, *M. L. Fonseca 2613* (UB). Minas Gerais: Vão do Paranã, Sept 1818, *C. F. P. von Martius 1793* (M, holotype, seen in digital image).

5.1.8. *Mimosa poculata Barneby*

BRAZIL. Tocantins: Palmeirópolis, Estrada de Acesso a Torre de Rádio de Enerpeixe, 16 Jun 2006, *G. Pereira-Silva* et al. *10743* (CEN62991).

5.1.9. *Mimosa somnambulans Barneby*

BRAZIL. Goiás: A 25 km SW de Monte Alegre de Goiás, 12 Jun 1973, *W. R. Anderson* et al. *6892* (UB1847, holotype; NY, R, isotypes); Nova Roma, estrada vicinal entre a GO-118 (Monte Alegre) e Nova Roma, km 11, 01 Jun 2014, *M. F. Simon* et al. *2450* (CEN87462).

5.1.10. *Mimosa scaberrima Hoehne*

BRAZIL. Mato Grosso: Cataqui–Iamain, Dec 1918, *J. G. Kuhlmann 2033* (NY1347018, isotype).

5.1.11. *Mimosa somnians Humb. & Bonpl. ex Willd.*

5.1.11.1. *Mimosa somnians* subsp. *lasiocarpa* (Benth.) Barneby

5.1.11.1.1. *Mimosa somnians* subsp. *lasiocarpa* var. *lasiocarpa*

BRAZIL. Goiás: Ad Rio Agapité, no date, *Claussen s.n.* (K, lectotype designated by Barneby (1991)); Cachoeira Dourada, na estrada de terra chegando na balsa que atravessa o rio Parnaíba entre Cachoeira Dourada e o rio, 09 Feb 2013, *J. E. Q. Farias 3169* (CEN89204); Corumbá de Goiás, topo do Pico dos Pirineus, Serra do Catingueiro, 6 km de Cocalzinho, 14 Jan 1981, *E. Nogueira 58* (NY935817, UB); GO 446, 42 km NW de Iaciara, camino a Nova Roma, 03 Feb 1990, *M. M. Arbo* et al. *3549* (BAB); Niquelândia, CODEMIN, ca. 40 km de Niquelândia, 15 Dec 1995, *M. L. Fonseca* et al. *736* (BAB). Mato Grosso: Fazenda Matão, 08 Feb 2016, *G. Pereira-Silva* et al. *16739* (CEN100328). Minas Gerais: Coromandel, Rod. MG188, Coromandel-Patrocínio. 18 km do trevo de saída em Coromandel, margem direita de estrada, 28 March 2013, *Borges* et al. *1028* (CEN98023); Ituiutaba, próximo a Ituiutaba, 10 Feb 2013, *J. E. Q. Farias* et al. *3180* (CEN89207); MG 188, 18 km al S de Paracatu, camino a Guarda Mor, 31 Jan 1990, *M. M. Arbo* et al. *3176* (BAB); MG 188, 28 km al S de Paracatu, cerca del rio Escurinho, camino a Guarda Mor, 31 Jan 1990, *M. M. Arbo* et al. *3209* (BAB); São Romão, 16 Dec 2000, *B. M. Gomes* et al. *127* (UB); Unaí, Ca. 1 km a montante do Eixo da Barragem, margem direita do rio Arrependido, 27 March 2002, *G. Pereira-Silva* et al. *6291* (CEN44847). Tocantins: Arraias, Aproximadamente 50 km de Arraias, estrada para Palmas, 02 Feb 1999, *M. F. Simon & J. C. M. Lima 41* (UB8108). Paraguay. Amambay: Prope Esperanza, Sierra de Amambay, no date, *E. Hassler 10683* and *10683a* (G, syntypes).

5.1.11.1.2. *Mimosa somnians* subsp. *lasiocarpa* var. *lupulina* (Benth.) Barneby

BRAZIL. Goiás: Niquelândia, ao longo da GO535, 6 km N de Niquelândia, 28 May 2014, *M. F. Simon* et al. *2436* (CEN87448); Macedo, Km 18 da estrada de chão para a mina de níquel, 14 Dec 1995, *M. L. Fonseca* et al. *718* (BAB); S of Crixas, no date, *Pohl 1413* (W, lectotype designated by Barneby (1991), seen in digital image). Minas Gerais: Grão Mogol, Beira da estrada, baixada entre lagoa intermitente com cerrado adjacente, 26 May 2005, *A. C. Sevilha* et al. *4505* (CEN61687).

5.1.11.2. *Mimosa somnians* subsp. *longipes* Barneby

5.1.11.2.1. *Mimosa somnians* subsp. *longipes* var. *longipes*

BRAZIL. Bahia: 7 km S of Rio Piauí, ±150 m SW of Barreiras, 13 Apr 1966, *H. S. Irwin* et al. *14697* (UB, holotype; K, NY, R, isotypes). Tocantins: Paraná, Fazenda Alegre, 15 May 2007, *G. Pereira-Silva* et al. *11816* (CEN71562).

5.1.11.2.2. *Mimosa somnians* subsp. *longipes* var. *possensis* Barneby

BRAZIL. Goiás: Posse, Serra Geral de Goiás, Rio da Prata, ca. 6 km S of Posse, 06 Apr 1966, *H. S. Irwin* et al. *14434* (NY957643); Posse, Serra Geral de Goiás, Rio da Prata, vicinity of Posse, 10 Apr 1966, *H. S. Irwin* et al. *14586* (NY3059).

5.1.11.3. *Mimosa somnians* subsp. *somnians*

5.1.11.3.1. *Mimosa somnians* subsp. *somnians* var. *deminuta* Barneby

VENEZUELA. Amazonas: Atures, carretera Puerto Ayacucho hacia El Burro; km. 28, a 6 km de la desviación hacia el E; Raudal del Agua Linda, 30 Apr 1993, *A. Gröger 863* (NY1347182). Bolívar: Cedeño, planicie aluvial, piedemonte y altiplanicie entre el margen derecho del Río Orinoco medio

y el borde NW de la Serranía de los Pijiguaos (Bajo Río Suapure); 2 km al NE de "La Guabina", 29 Oct 1987, *F. J. Guánchez & O. Huber 4782* (NY1347184); Piedra Marimare, E bank of Río Orinoco, opposite heead of Isla El Gallo, 19 Dec 1955, *J. J. Wurdack & J. V. Monachino 40850* (NY3056, holotype).

5.1.11.3.2. *Mimosa somnians* subsp. *somnians* var. *somnians*

MÉXICO. Chiapas: Cintalapa, near the microwave station of La Mina, 12 km S of Mexican Highway 190, near Rizo de Oro, 16 Oct 1971, *D. E. Breedlove & R. F. Thorne 20572* (NY551397). Nayarit: Nayarit, 13 mi SE of turn-off to San Blas on Hwy 15, 09 Apr 1970, *W. F. Mahler* et al. *5876* (NY551408); Tepic, 8 mi SW of Tepic, 08 Apr 1970, *W. F. Mahler* et al. *5874* (NY551409). Oaxaca: Totontepec Villa de Morelos. Mixes, 01 Nov 1987, *E. Velazco López 92* (NY459055). GUATEMALA. Santa rosa: Along CA-1 highway, 10.7 mi. W of Ciulapa, 26 Jul 1978, *C. Dziekanowski* et al. *3157* (NY1452760). HONDURAS. Colón: Capuchin site east; 1.8 mi. strip on the north bank of río Guaimoreto, between old bridge and opening of Laguna Guaimoreto, 4.5 mi. NE of Trujillo on old road to Castilla, 27 Jan 1981, *J. G. Saunders 943* (NY1452758). EL SALVADOR. Santa Ana: Vicinity of Santa Ana, 28-30 Jan 1922, *P. C. Standley 20382* (NY1452761). NICARAGUA: Zelaya, Less than a km NW of Puerto Cabezas, 13 Jul 1970, *G. Davidse & R. W. Pohl 2328* (NY1472762). COSTA RICA. Alajuela, Del Cacao de Alajuela, 11 Dec 1932, *A. M. Brenes s.n.* (NY1452766). PANAMÁ. Coclé: Along roadside, ca. 12 km by road NE of Rio Hato along highway to Panama City, 18 Jun 1974, *A. A. Lasseigne 4292* (1452772). TRINIDAD AND TOBAGO: Prope Scarborough, Dec 1913, *W. E. Broadway 4776* (NY, isotype of *M. tobagensis* Urban). COLOMBIA. Cundinamarca: A midway between Sasaima and Villeta, ca. 60 km NW of Bogotá, 25 Jun 1974, *A. A. Lasseigne 4302* (NY1347146); Meta, San Juan de Arama, Estación "La Curia", Reserva Natural de La Macarena, entre la estación y el río Guejar, 24 Sept 1987, *J. Estrada & J. L. Fernández 352* (NY1347118); Santander, northern slope of Mesa de Los Santos, 11-15 Dec 1926, *E. P. Killip & A. C. Smith 15425* (NY1347142). Tolima: Cuesta de Tolima, no date, *Humboldt & Bonpland 1845* (P, isotype, seen in digital image). BRAZIL. Goiás: Minaçu, Estrada entre a UHE da Serra da Mesa e Minaçu, km. 3, 10 Jun 1999, *M. F. Simon 282* (UB). Mato Grosso: Rodovia Campo Grande-São Paulo, km 25, 10 Jun 1976, *H. F. Leitão Filho 2116* (CEN2701). Minas Gerais: Between Pirapora and Genipapo, Jul 1820, *Pohl 1428* (NY, isolectotype of *M. podocarpa* Benth.). Tocantins: Ponte Alta, Estrada para Monte de Carmo, ca. de 30 km de Ponte Alta do Tocantins, 18 Jul 2000, *V. C. Souza* et al. 24189 (CEN85948). BOLIVIA**.** Santa Cruz: Ichilo, 3 km al N de Buena Vista, sobre el camino, 19 May 1991, *R. H. Fortunato & A. Krapovickas 1910* (BAB). PARAGUAY. Caaguazú: Caaguazú, 26 Feb 2001, *E. M. Zardini & L. Guerrero 56011* (BAB). Canindeyú: Camino a Jejuí Mi, 16 May 2003, *S. Sede 71* (BAB). Central: Tavarory, 1 km SE from entrance, 16 Jun 1994, *E. M. Zardini & M. Vera 39501* (BAB). Concepción: Camino a Paso Mbutú, Karaza Bola, 18 Dec 1986, *R. H. Fortunato* et al. *848* (BAB). Cordillera: 5,5 km por Ruta 3 al S de Emboscada, 14 Mar 2005, *R. H. Fortunato* et al. *8801* (BAB); Tobatí, Cerro Mirador, 06 Mar 2008, *R. H. Fortunato* et al. *9170* (BAB); Tobatí, "Ybytú Silla" mesa, middle area, 23 Feb 1991, *E. M. Zardini & C. Velásquez 26587* (BAB). Paraguarí: Metros de la casa del puestero de la Estancia Ypoá, en dirección a la ruta, 07 Mar 2005, *R. H. Fortunato* et al. *8527* (BAB). San Pedro: Salida de San Estanislao en dirección a Itacurubí del Rosario, 500 m de la rotonda por Ruta 3, 07 Mar 2008, *R. H. Fortunato* et al. *9194* (BAB). ARGENTINA**.** Misiones: Capital, Posadas, 12 Jan 1907, *C. Spegazzini s.n.* (BAB 18350); Refugio Don Lorenzo, 24 May 1997, *A. Honfi* et al. *798* (BAB). Corrientes: Berón de Astrada, Tuyuty, 03 May 1945, *A. M. Ruiz Huidobro 2141* (BAB 70713); Gral. Alvear: 12 km al N de Alvear, por Ruta Nacional 14, 11 Mar 2006, *R. H. Fortunato* et al. *9096* (BAB); Ituzaingó, 17,5 km al N por Ruta Prov. 150 en dirección a Ombú Chico, 02 Feb 2003, *R. H. Fortunato* et al. *7953* (BAB); Camino a la Represa Yaciretá, 15 Feb 2008, *M. Morales* et al. *653* (BAB).

5.1.11.4. *Mimosa somnians* subsp. *viscida* (Willd) Barneby

5.1.11.4.1. *Mimosa somnians* subsp. *viscida* var. *aquatica* Barneby

Brazil. Mato Grosso: São Antonio de Leverger, near São Antonio de Leverger, 15-25 km S of Cuiabá, 12 Feb 1975, *W. R. Anderson* et al. *11350* (MBM, holotype; NY, UB, isotypes).

5.1.11.4.2. *Mimosa somnians* subsp. *viscida* var. *leptocaulis* (Benth.) Barneby

BRAZIL. Goiás: Ad Morro de Manuel Gomes, Mar 1819, *Pohl 1379=1477* (K, holotype); Monte Alegre, ao longo da GO118 20 km ao S de Monte Alegre, 03 Jun 2014, *M. F. Simon* et al. *2449* (CEN87461). Tocantins: Goiatins, Folha SB-23-Y-C, Bacia do Tocantins, sub-bacia Rio Tocantins, 31 Mar 2010, *R. F. Haidar 1294* (CEN86177); Mateiros, Cachoeira da Velha, 11 May 2001, *C. E. B. Proença* et al. *2556* (UB); Mateiros, 03 May 2001, *R. Farias 366* (CEN51498).

5.1.11.4.3. *Mimosa somnians* subsp. *viscida* var. *viscida*

BRAZIL. Pará: no locality, no date, *Willdenow B-WILLD19087* (B-WILLD, holotype, seen in microform). Goiás: Calcavante, Reserva Natural Serra do Tombador, 13 May 2014, *A. T. Fidelis* et al. *914* (CEN91739); Corumbaíba: Margem esquerda do Río Corumbá, 2 km a montante do Eixo da Barragem, *H. C. P. dos Santos* et al. *38* (CEN); Damianópolis, Entrada vicinal a 10 km W de Damianópolis, 01 March 2014, *M. Simon* et al. *2087* (CEN); Goiás Velho, Serra Dourada, 18 Dec 1968, *G. M. Barroso 800* (NY957655, UB); Goiás Velho, Serra Dourada, no date, *J. E. Pohl 1376=1759* (K, lectotype of *M. trijuga* Benth. designated by Barneby (1991)); Minaçu, Reserva da Serra da Cana Brava, 08 Jun 1995, *R. I. P. de Freitas* et al. *6* (UB); Pirenópolis, ao longo da GO-338, 17 km N do Pirenópolis, trilha 2 km NW de Cachoeira das Araras, 29 March 2015, *M. Simon* et al. *2597* (CEN). Maranhão: P. N. Chapada das Mesas, acesso E no km 612 da BR130, 20 km em estrada vicinal em direção à localidade Buenos Aires, 07 Apr 2016, *M. F. Simon* et al. *2778* (CEN).

5.1.11.4.4. *Mimosa somnians* subsp. *viscida* var. *velascoënsis* (Harms) Barneby

BRAZIL. Goiás: Calcavante, Margem direita do Rio Tocantins, ca. 800 m da Balsa Serra Branca (COTERRA), 17 Apr 2001, *G. Pereira-Silva* et al. *4943* (CEN42137). Tocantins: Jalapão, Norte, beira da Estrada, 07 May 2001, *L. H. Soares e Silva 906* (CEN41612); Paranã, margem direita do lago da UHE São Salvador, 26 Mar 2009, *G. Pereira-Silva 14165* (CEN84302).

5.1.12. *Mimosa surumuënsis Harms*

GUYANA. Pakaraima mountains, Upper Ireng River watershed; Malakwalai–Tipu summit along SE escarpment Potaro–Siparuni, 13 Jul 1994, *T. W. Henkel 5574* (NY1347232). VENEZUELA. Frequent on Chinese slide on lower northern slope of Cerro Bolívar, 24 Oct 1963, *B. Maguire 35963* (NY1347225). BRAZIL. Roraima: Surumu, Serra do Mel, Rio Branco, Jul 1909, *E. H. G. Ule 8131* (NY3084, RB, isotypes).

5.1.13. *Mimosa trinerva V.F.Dutra & F.C.P.Garcia*

BRAZIL. Minas Gerais: Rio Pardo de Minas, trilha em direção a casa de apoio, Parque Estadual de Serra Nova e Talhado, 28 Aug 2019, *A. Nepomuceno* et al. *956* (VIES45211); Rio Pardo de Minas, Parque Estadual Serra Nova, trilha até o Gerais de Santana, 22 Mar 2012, *J. A. Lombardi* et al. *9073* (CEN94130).
